# Polyphenols and IUGR Pregnancies: Effects of Maternal Hydroxytyrosol Supplementation on Placental Gene Expression and Fetal Antioxidant Status, DNA-Methylation and Phenotype

**DOI:** 10.3390/ijms20051187

**Published:** 2019-03-08

**Authors:** Consolación Garcia-Contreras, Marta Vazquez-Gomez, Alicia Barbero, José Luis Pesantez, Angelo Zinellu, Fiammetta Berlinguer, Pedro Gonzalez-Añover, Jorge Gonzalez, Teresa Encinas, Laura Torres-Rovira, Yolanda Nuñez, Jaime Ballesteros, Miriam Ayuso, Susana Astiz, Beatriz Isabel, Cristina Ovilo, Antonio Gonzalez-Bulnes

**Affiliations:** 1 Instituto Nacional de Investigación y Tecnología Agraria y Alimentaria (INIA), Ctra. de La Coruña Km. 7,5, 28040 Madrid, Spain; garcia.consolacion@inia.es (C.G.-C.); jose.pesantez@ucuenca.edu.ec (J.L.P.); torres.laura@inia.es (L.T.-R.); nunez.yolanda@inia.es (Y.N.); jaime.ballesteros@inia.es (J.B.); astiz.susana@inia.es (S.A.); ovilo@inia.es (C.O.); 2 Faculty of Veterinary Medicine, Universidad Complutense de Madrid (UCM), Ciudad Universitaria s/n, 28040 Madrid, Spain; mvgomez@ucm.es (M.V.-G.); pedrogonzalez@cuartesa.com (P.G.-A.); tencinas@vet.ucm.es (T.E.); bisabelr@vet.ucm.es (B.I.); 3 Diagnostic Imaging Service, Universidad Alfonso X El Sabio, 28691 Madrid, Spain; aliciabarbero.vet@gmail.com; 4 School of Veterinary Medicine and Zootechnics, Faculty of Agricultural Sciences, University of Cuenca, Avda. Doce de Octubre, 010220 Cuenca, Ecuador; 5 Department of Biomedical Sciences, University of Sassari, Viale San Pietro 43/B, 07100 Sassari, Italy; azinellu@uniss.it; 6 Department of Veterinary Medicine, University of Sassari, Via Vienna 2, 07100 Sassari, Italy; berling@uniss.it; 7 Micros Veterinaria, Campus de Vegazana, 24007 Leon, Spain; info@microsvet.es; 8 Laboratory of Applied Veterinary Morphology, Department of Veterinary Sciences, Faculty of Biomedical, Pharmaceutical and Veterinary Sciences, University of Antwerp, 2610 Wilrijk, Belgium; Miriam.AyusoHernando@uantwerpen.be

**Keywords:** antioxidants, intrauterine-growth-restriction, pregnancy, swine-model

## Abstract

The use of polyphenols is a promising strategy for preventing or alleviating intrauterine growth restriction (IUGR) because polyphenol supplementation increases plasma antioxidant capacity and improves oxidative stress at the feto-placental unit; which are recognized as main issues in IUGR. However, there is a scarcity of experimental data on both realistic benefits and potential hazards of polyphenol supplementation during gestation. Hence, we aimed to use a swine model of IUGR pregnancy to determine possible effects of maternal supplementation with polyphenols (hydroxytyrosol) on placental expression of genes involved in antioxidant homeostasis, vascularization and fetal growth and thus on antioxidant status, DNA-methylation and phenotypic traits (morphology and homeostasis) of the fetus. Hydroxytyrosol improves placental gene expression and fetal antioxidant status and glucose metabolism in a sex-dependent manner, in which males were favored in spite of developmental failures. Concomitantly, hydroxytyrosol prevented hypomethylation of DNA associated with oxidative stress. Finally, no major deleterious effects of hydroxytyrosol supplementation on constriction of the ductus arteriosus, a possible secondary effect of polyphenols during pregnancy, were found.

## 1. Introduction

Hydroxytyrosol is a polyphenol present in olive leaves and fruits (and, hence, in virgin olive oil) with prominent antioxidant, metabolism-regulatory, anti-inflammatory and immuno-modulatory properties [1]. There is increasing clinical and epidemiological evidence of its relevance against different pathologies (e.g., cancer, cardiovascular, metabolic and neurodegenerative diseases [2]) and, recently, our group showed its usefulness as a maternal supplement during pregnancy to counteract the appearance of intrauterine growth restriction (IUGR), and therefore appearance of low-birth-weight (LBW) neonates in a swine model [3].

The reason for using hydroxytyrosol, or other polyphenols, in pregnancies at risk of IUGR is due to the fact that the disease is primarily related to an impaired supply of nutrients and oxygen and, consequently, a weakened antioxidant defense system [4,5,6,7]. Polyphenol supplementation increases plasma antioxidant capacity [8,9] and improves placental oxidative stress [10]. In this regard, it is well established that improving the oxidative stress status during pregnancy is related to alleviation of the IUGR process [11,12,13]. Hence, consumption of hydroxytyrosol-rich food, like olive oil, may be beneficial, on the whole, for pregnancy and, specifically, for IUGR-compromised pregnancies. However, in a similar way to other polyphenol compounds, there is a scarcity of experimental data on both the realistic benefits and potential hazards of hydroxytyrosol supplementation during gestation [9,14,15] despite the promising results obtained in our model [3].

There is, in fact, some evidence suggesting polyphenols have deleterious effects during pregnancy. The main concern is related to their anti-inflammatory actions, which, in a similar way to some anti-inflammatory drugs, may trigger changes in the fetal vascular system; specifically, constriction of the ductus arteriosus [16]. The ductus arteriosus drives the fetal blood flow from the right ventricle to the descending aorta and its constriction/obliteration may cause complications such as fetal and neonatal heart failure, hydrops and neonatal pulmonary hypertension, which may cause death [17,18,19]. A second concerning issue for the use of polyphenols during pregnancy is the existence of evidence pointing to the induction of epigenetic changes by DNA-methylation [2]; most of them are adaptive and beneficial, but deleterious effects cannot be discarded. Clarification of these points is essential for issuing dietary guidelines during pregnancy and, therefore, we aimed to use our swine model to determine possible effects of maternal hydroxytyrosol supplementation on placental expression of genes involved in antioxidant homeostasis, vascularization and fetal growth and thus on antioxidant status, DNA-methylation and phenotypic traits (morphology and homeostasis) of the fetus.

## 2. Results

The assessment of maternal features in the groups C and HTX at sampling (Table 1) showed no significant differences in body weight, back-fat depth, plasma antioxidant status (total antioxidant capacity in terms of Trolox Equivalent Antioxidant Capacity, TEAC, and concentrations of glutathione, glycine and taurine), plasma parameters of metabolism of glucose (glucose, fructosamine), and lipids (triglycerides and total-, HDL- and LDL-cholesterol).

A total of 55 and 45 fetuses were obtained, respectively, in the control (group C) and hydroxytyrosol-treated group (group HTX). Hence, the mean litter size was similar in both groups (7.8 ± 0.7 for C vs. 7.5 ± 1.2 for HTX). The sex ratio of the piglets was also similar and close to 1:1 in both groups; 31 fetuses were females and 24 were males in the group C (56.4 vs. 43.6%) while 26 fetuses were females and 19 were males in the group HTX (57.8 vs. 42.2%).

### 2.1. Maternal and Fetal Plasma Hydroxytyrosol Concentrations

Plasma hydroxytyrosol concentrations were almost undetectable, in both the sows and their fetuses, after fasting for approximately 16 h. Only one sow (16.7%) and six fetuses (13.3%) showed detectable levels of the substance (0.54 μg/mL and 0.46 ± 0.06 μg/mL, respectively).

### 2.2. Placental Candidate Gene Expression

**Figure 1:**
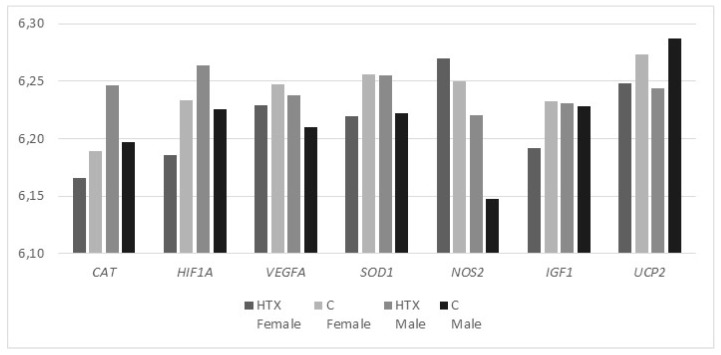
Relative gene expression of antioxidant, vascularization and fetal growth candidate genes in placenta of Iberian fetus from sows supplemented with hydroxytyrosol (HTX) and controls (C).

Treatment, gender and interaction treatment*gender effects on candidate gene expression were analyzed. No significant effect was observed for treatment or gender effects for any gene. Regarding the interaction treatment*sex, significant effects were found for *SOD1* and *HIF1A* genes (*p* = 0.008 and 0.04 respectively). Males subjected to antioxidant treatment showed higher expression than females for both genes, while the opposite was observed in the control group, with females showing upregulation of *SOD1* and *HIF1A* (Figure 1). The same pattern was observed for *CAT* and *VEGFA* genes, although these genes did not reach statistical significance.

The expression values obtained for *CAT*, *VEGFA*, *HIF1A*, *SOD1* and *NOS2* genes were highly correlated (0.34≤ r ≤0.81; 0.03≤ *p* ≤0.0001). The *CAT* gene was negatively correlated to fetal weight (*r* = −0.5, *p* = 0.001).

### 2.3. Fetal Antioxidant Status

The assessment of antioxidant status (Table 2) showed a higher total antioxidant capacity (TEAC) in fetuses from the group HTX than in fetuses from the control group C (*p* < 0.0001). The values for TEAC were affected by sex in a different way in the group C and HTX. Female fetuses had higher values than males in the group C (*p* < 0.0005) whilst values were higher in males of group HTX (*p* < 0.05).

Plasma concentrations of glutathione, glycine, serine and taurine were similar in both groups. There were no significant effects from sex in the group C, but males in group HTX showed a numerically higher glutathione concentrations (*p* = 0.07) and significantly lower concentrations of serine (*p* < 0.001) and glycine (*p* < 0.05) than their female counterparts.

### 2.4. Fetal Development

The comparison of body measurements between fetuses in control and treated groups showed no significant differences in lengths and widths of body and head (Table 3). On the other hand, fetuses in the group HTX showed lower mean body weight (*p* < 0.005) and lower mean weights of total viscerae, lungs, liver, pancreas and intestine (*p* < 0.05) than fetuses in the group C.

These differences were driven by sex-related effects. There were no significant differences in any of the parameters when comparing females from groups C and HTX, but males HTX had lower values than males C for biparietal diameter and thoracic circumference (*p* < 0.01 for both), body-weight (*p* < 0.05) and weights of total viscerae and liver (*p* < 0.05 for both) and intestine (*p* < 0.001). There were no significant effects from fetal sex in either group.

Analysis of weight ratios among different organs and total body weight showed that HTX fetuses had significantly higher values for brain/body-weight and kidneys/body-weight (*p* < 0.05). There were no significant differences when comparing females and males within groups or females HTX and females C, but HTX males had higher ratios for brain/body-weight and heart/body-weight (*p* < 0.05) than C males.

### 2.5. Morphological Appearance of Fetal Descending Aorta and Ductus Arteriosus

**Figure 2:**
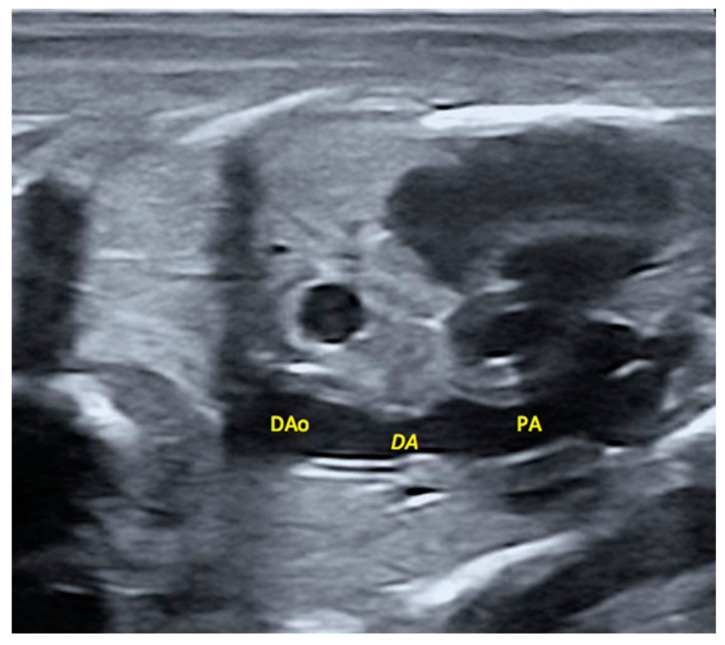
This image shows an apparent narrowing of the ductus arteriosus. DAo: Descending Aorta; DA: Ductus arteriosus; PA: Pulmonary Artery.

The ultrasonographic assessment of the diameter of the descending aorta showed higher mean values in the group HTX than in the group C (3.5 ± 0.2 vs. 2.9 ± 0.2 mm, respectively; *p* = 0.01), whilst differences did not reach statistical significance for the ductus arteriosus (2.8 ± 0.2 for group HTX vs. 2.4 ± 0.2 for group C; *p* = 0.08). The assessment of the ratio ductus arteriosus/descending aorta showed similar values in both groups (0.8 ± 0.1 for group HTX vs. 0.8 ± 0.1 for group C). There were no significant effects from sex. The group C had 6.3% of fetuses with a ratio 1 SD below the mean ratio whilst this percentage reached 17.4% in the group HTX, with half of these fetuses in the group HTX showing a ratio 2 SD lower than the mean and anatomical evidence of ductal narrowing (Figure 2). However, differences were not statistically significant.

### 2.6. Fetal Muscle Fibers

The comparison of the number of secondary fibers and the area of the muscle fiber between control and treated fetuses showed no overall significant differences (Table 4). However, there was a significant interaction between treatment and sex. In the group C, males had both a lower number of secondary fibers and a smaller area of the fiber (*p* < 0.005 for both). Conversely, there were no significant differences between male and female fetuses in the group HTX, where females had similar values to females from the group C, whilst males from group HTX showed a significant increase when compared to males in the group C (*p* < 0.005 for secondary fibers and *p* < 0.005 for fiber area).

### 2.7. Fetal Metabolic Status (Glycemic and Lipid Profiles)

The analysis of plasma concentrations of parameters for glycemic (glucose, fructosamine) and lipid profile (triglycerides, total cholesterol, HDL-c and LDL-c) is shown in Table 5. Significantly lower values in the group HTX than in the group C for glycemic indexes (*p* < 0.05 for glucose and *p* < 0.01 for fructosamine) were found but no difference was found for lipid indexes. Differences in glycemic indexes were again conditional on sex. There were significantly higher values for fructosamine in males HTX than in males C (*p* < 0.01).

### 2.8. Fetal DNA Methylation

The analysis of DNA methylation showed significantly higher values in the group HTX than in the group C (4.3 ± 0.1 vs. 4.0 ± 0.1, respectively; *p* < 0.01), without sex-related effects. There were no significant differences in the ratio of serine to glycine between groups HTX and C (0.38 ± 0.1 vs. 0.37 ± 0.1) but HTX females had higher values than HTX males (0.40 ± 0.1 vs. 0.36 ± 0.1, respectively; *p* < 0.05) and C females (0.37 ± 0.1; *p* = 0.07).

## 3. Discussion

The present study is, to the best of our knowledge, the first attempt to elucidate the effects (positive or deleterious) of maternal hydroxytyrosol supplementation on antioxidant and metabolic status, DNA-methylation, and developmental and morphological patterns of fetuses at risk of growth retardation.

First, the current study indicates that the effects of maternal hydroxytyrosol supplementation are more related to the triggering of a cascade of events rather than to direct effects of the substance itself. There was either no major transfer of hydroxytyrosol to the fetuses or hydroxytyrosol was cleared after approximately 16 h of maternal fasting; in any case, fetuses were not permanently exposed to hydroxytyrosol. However, there was a significant effect of maternal hydroxytyrosol supplementation on the improvement of the fetal antioxidant status. Fetuses from the treated group (group HTX) showed a significantly higher total antioxidant capacity (TEAC) and a trend towards higher glutathione concentrations than that of the fetuses from the control group (group C), supporting previous data indicating that offspring exposed to polyphenols have increased antioxidant activity [10,20,21].

Second, the results obtained in this study indicate that the effects of maternal hydroxytyrosol supplementation are quite different depending on the sex of the offspring. The influence of fetal sex is a constant feature in studies regarding fetal growth restriction and prenatal programming, as reviewed by Mortiz et al. (2010) [22] and Aiken and Ozanne (2013) [23]. In this sense, the current data are in line with observational studies in humans [24,25] and experimental data in animal models [26,27]. Our study indicates, for the first time, that the effects from hydroxytyrosol supplementation on fetal antioxidant status are driven by the sex of the offspring. In the group HTX, male fetuses showed higher TEAC values (as opposed to the group C where females showed higher values than males) and glutathione values. Conversely, glycine and serine concentrations were higher in females.

Glutathione is an important antioxidant agent that prevents cellular damage caused by reactive oxygen species (mainly free radicals and peroxides, specifically, lipid peroxides [28]) and its role in lowering oxidative stress has been also demonstrated in newborns piglets with growth retardation [29]. Glutathione is synthesized from glycine, which is also a potent antioxidant agent and metabolic regulator and which, in turn, is synthesized from serine [30]. Hence, the higher glutathione levels and lower levels of its precursors in males compared to females of the group HTX, without significant differences in the group C, allows us to hypothesize that the higher antioxidant capacity induced by hydroxytyrosol supplementation in males, besides the activation of superoxide dismutase (SOD), relies on the enhancement of the glutathione route, as previously described in adult cells [31].

Over the recent years, a significant role of glutathione metabolism on epigenetic regulation, through increasing DNA methylation, has been identified [32]. Concomitantly, data from the present study show that DNA methylation increased in the fetuses from the group HTX when compared to those from the group C, although there were no sex-related differences within groups in this case. The higher ratio of serine to glycine found in the females of the group HTX is evidence for the role of methylneogenesis for DNA re-methylation, counteracting the DNA hypomethylation characteristic of oxidative stress; in fact, reductions in markers of oxidative stress correlate with increases in DNA methylation [33,34]. Hence, these results pave the way for further studies on the significance and role of such changes in DNA methylation as hyper- or hypo-methylation induced by diet may be beneficial for some genes, but deleterious for the normal expression of other genes [34].

However, in spite of the evidence supporting the effects of hydroxytyrosol supplementation on their antioxidant status and DNA methylation, the growth of the male HTX fetuses was penalized when compared to control counterparts and female littermates.

In this sense, the assessment of sex-related effects of maternal nutritional restriction on fetal features in the control fetuses (group C) showed that there was a trend towards a higher body-weight and corpulence in male fetuses compared to female. Such a finding is in agreement with previous studies in younger fetuses (Days 70–90 instead of Day 100 in the present study [35,36]) and newborns [3,37] of the same breed and nutritional management. Surprisingly, this larger body development in males was accompanied by worse muscle development, as evidenced by a smaller fiber area and a lower number of secondary fibers than their female littermates. Previous studies indicate that piglets with growth retardation have a clear restriction of muscle development [38,39,40]; however, we have not found information on sex-related differences.

In opposition, males in the group HTX showed similar weights to their female littermates and lower than weights of males from the group C. There were no differences between females in the groups C and HTX, so these findings suggest a sex-related penalization of the growth in males HTX. In agreement, placental HIF1A gene expression, which has been shown to be activated in the placenta of growth restricted fetuses [41], showed the highest expression level in HTX males. In addition, CAT gene expression was negatively correlated to fetal weight, suggesting a placental antioxidant response to impaired fetal growth.

The influence of males determined that, ultimately, there were no significant differences in body corpulence (measurements) between groups C and HTX but, overall, fetuses in the group HTX showed lower total body-weight and lower weights of some viscerae (lungs, liver, pancreas and intestine). These results are, at a first glance, opposite to our own findings in newborns [3], in which the group HTX was characterized by a higher birth-weight than the group C, without statistically significant differences between females in the groups C and HTX but higher birth-weights in HTX compared to C males. The positive effects from hydroxytyrosol supplementation on neonate birth-weights have been confirmed by a subsequent study in hyperprolific sows (unpublished results). We do not have a clear explanation for such opposite results from the data of the present trial, but differences among studies may be caused by the fact that the weight of the fetuses was assessed in this trial at Day 100 of pregnancy. It is well-known that fetal development follows an exponential curve, increasing quadratically from Day 45 to 115 of pregnancy with the greatest increase occurring during the last 15 days of gestation [42]. The better antioxidant status of the fetuses at Day 100 of gestation might exert a beneficial effect in the last rapid growth phase, with a more efficient utilization of nutrients; hence, a heavier birth weight for the group HTX than for controls could be possible. On the other hand, inconsistencies in the effects of hydroxytyrosol supplementation cannot be overlooked. Further experiments would therefore be necessary to clarify the differences among studies.

In fact, the results found in the males of the present trial give rise to different questions that need to be addressed in subsequent research. In the current study, the male HTX fetuses had a lower body-weight; however, they showed better indexes of antioxidant status and glucose metabolism (evidenced by significantly higher fructosamine concentrations, a good index for long-period glycaemia since it represents the average glucose during previous days) and activation of placental antioxidant defense genes. The male HTX fetuses also showed better compensatory growth patterns (with higher values for the relative weights of brains and kidneys and heart to body-weight), and a higher area of muscle fiber and more secondary fibers than their control male counterparts.

Finally, the assessment of possible constrictions of the ductus arteriosus did not prove alarming evidence of fetuses with ductal narrowing. In any case, the appearance of some fetuses with ductal narrowing in the group HTX, although it did not reach statistical significance when compared to group C, may deserve further studies to clarify its clinical significance.

## 4. Methods

### 4.1. Ethics Statement

The experimental procedures were all approved by the Instituto Nacional de Investigación y Tecnología Agraria y Alimentaria (INIA) Committee of Ethics in Animal Research (report CEEA 2013/036) and performed at the INIA animal facilities, in agreement with the Spanish Policy for Animal Protection RD53/2013 which complies with the European Union Directive 2010/63/UE on the protection of animals used for research.

### 4.2. Animals and Experimental Procedures

The study involved 13 primiparous Iberian sows, which became pregnant after cycle synchronization with altrenogest (Regumate^®^, MSD, Boxmeer, The Netherlands) and insemination with cooled semen from a purebred Iberian boar. These sows were fed with a standard grain-based food diet with the following mean component values: dry matter, 89.8%; crude protein, 15.1%; fat, 2.8%; and metabolizable energy, 3.0 Mcal/kg.

The amount of food was adjusted to fulfill individual daily maintenance requirements from the start of the experimental period to Day 35 of pregnancy. On Day 35 of pregnancy, all the sows were weighed and the amount of food offered to each sow was adjusted to fulfill 50% of their daily maintenance requirements; a diet which has been previously found to impose a nutritional challenge and to affect fetal development inducing lower birth-weight in the newborns [37,43]. On the same day, the sows were pair-matched according to body-weight with seven females acting as an untreated control group (group C), whilst the other six females (group HTX) were treated by receiving 1.5 mg of hydroxytyrosol per kg of feed per day from Day 35 of pregnancy to day of sampling (Day 100 of pregnancy).

Day 100 of pregnancy, corresponding to approximately 90% of a 112-day gestation length typical for this breed, was chosen because, from Day 90 onwards, fetal metabolism becomes independent from maternal signals and is highly affected by nutrient availability [44].

At Day 100, sows were weighed, back-fat depth was evaluated at the right-side at 4 cm from the midline and the head of the last rib by ultrasonography (5–8 MHz lineal-array probe, SonoSite Inc., Bothell, WA, USA), and blood samples were drawn from the orbital sinus after fasting for approximately 16 h. Samples were collected in sterile, heparinized 4-ml vacuum tubes (Vacutainer™ Systems Europe, Meylan, France) and were immediately centrifuged at 1500× *g* for 15 min. The plasma was separated and biobanked into polypropylene vials at −80 °C until they were assayed for hydroxytyrosol concentrations, homeostasis biomarkers (i.e., antioxidant status, glycemic values, and amino acids and lipid profiles) and DNA-methylation.

### 4.3. Measuring, Weighting and Sampling of Fetuses

**Figure 3:**
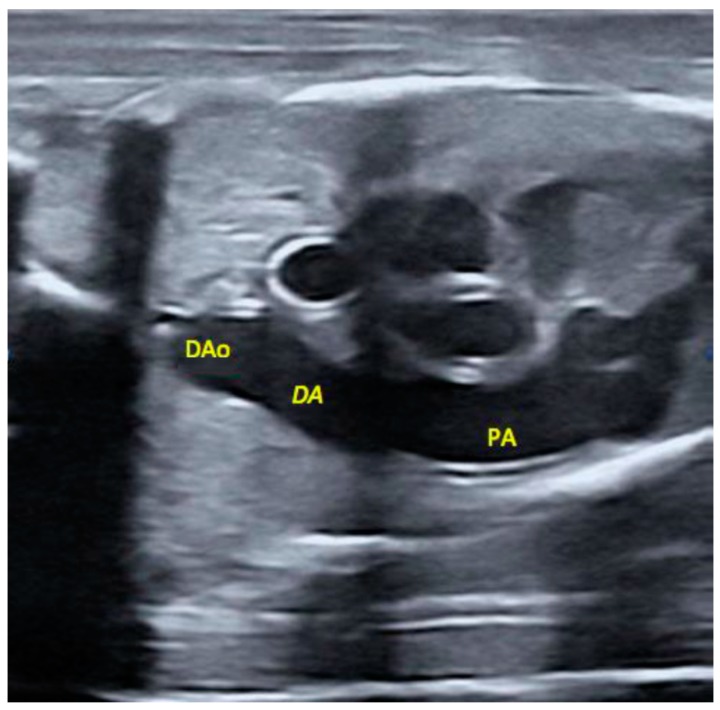
The image shows gray scale normal appearance of the ductus arteriosus. DAo: Descending Aorta; DA: Ductus arteriosus; PA: Pulmonary Artery.

The sows were sequentially euthanized by stunning and exsanguination, in compliance with RD53/2013 standard procedures. In each one, the entire genital tract was immediately collected for morphometric evaluation and fetal sampling. At once, conceptus position was recorded and all the fetuses were assessed by ultrasonography, with a Mylab Alpha ultrasound machine (Esaote, Genova, Italy) equipped with an automatic 2–5 MHz 4D convex probe, for measuring the diameters of the ductus arteriosus and descending aorta with built-in electronic calipers after video-recording of the structures (Figure 3).

Immediately, the content of the uterus was exposed and, for each fetus, blood samples were drawn from the heart using heparinized syringes and processed as described above for sows. Placenta samples were obtained and stored at −80 °C until their use for gene expression studies and fetuses were removed and sex was determined by visual inspection immediately after recovery. The entire fetuses were immediately weighed and measured. Measurements included crown-rump length (CRL), occipito-nasal length (ONL), biparietal diameter thoracic (BDP) and thoracic and abdominal circumferences (TC and AC, respectively). Afterwards, total thoracic and abdominal viscerae and brain, heart, lungs, liver, intestine, kidneys, spleen and pancreas were separated to determine their absolute weight and their relative weight to total body weight. Finally, a sample of longissimus dorsi muscle was fixed in 4% paraformaldehyde for evaluation of muscle fiber amount and distribution.

### 4.4. Evaluation of Maternal and Fetal Hydroxytyrosol Concentrations

Plasma hydroxytyrosol concentrations, in both maternal and fetal plasma, were determined by high performance liquid chromatography (HPLC). Plasma samples (250 µL each) were placed in glass test tubes and mixed with 50 μL of ascorbic acid (100 ng/mL) solution, 50 V of serine (200 ng/mL) solution and 2 mL of acetonitrile. The mixture was vigorously vortexed for 2 min and centrifuged at 1500× *g* for 10 min. The upper (organic) phase was collected and dried under a nitrogen atmosphere at 40 °C. The extracts were reconstituted in 250 µL of acetonitrile, vortexed, mixed for 20 s, and then transferred to autosampler vials for HPLC analysis.

Measurements were performed by a reversed-phase HPLC-UV system which consisted of Spectra-physic Series (Thermo Scientific, Essex, UK) components including a pump (P100), an autosampler (AS1000), and a variable fluorescence detector (UV100) set at 281 and 316 nm for excitation and emission, respectively. The detector signals were recorded with a Spectra physic integrator (DATAJET CH1). Separation was done in a Mediterranean sea-C_18_ column (Teknokroma, Barcelona, Spain; 150 mm × 4.6 mm i.d., 5 µm particle size) at 25 °C. The mobile phase was water:glacial acetic acid:acetonitrile (84:2:14 by volume; pH = 2.6) and was pumped at a constant flow rate of 1.0 mL/min. The estimated recovery of hydroxytyrosol after extraction procedures was 90.1%. The calibration curves showed a correlation coefficient of >0.996 and the limit of detection was 80 ng/mL.

### 4.5. Evaluation of Maternal and Fetal Antioxidant Status

The values for total antioxidant capacity, in terms of Trolox Equivalent Antioxidant Capacity (TEAC), were determined in both maternal and fetal plasma. TEAC was measured using the method described by Re et al. (1999) [45] and modified by Lewinska et al. (2007) [46]. Substances with antioxidant properties like taurine and glutathione, the most abundant low-molecular-weight antioxidants in animal tissues including the placenta [47], and their precursors, glycine and serine, were determined by capillary electrophoresis as previously reported [48,49].

### 4.6. Evaluation of Maternal and Fetal Metabolic Status

Parameters for glycemic index (glucose, fructosamine) and lipid profile (triglycerides, total cholesterol, high-density lipoproteins cholesterol [HDL-c] and low-density lipoproteins cholesterol [LDL-c]) were measured in maternal and fetal plasma. Assays were performed using a clinical chemistry analyzer (Saturno 300 plus, Crony Instruments s.r.l., Rome, Italy), according to the manufacturer’s instructions.

### 4.7. Evaluation of Placental Candidate Gene Expression

Total RNA was extracted from 50–100 mg of placental chorion obtained from 40 fetuses corresponding to both fetal genders and treatments (10 HTX-males, 10 HTX-females, 10 C-males and 10 C-females). RNA was extracted using RiboPureTM RNA isolation kit (Ambion, Austin, TX, USA) following the manufacturer’s recommendations. Samples were homogenized with 1 mL of TRI Reagent (Ambion, Austin, TX, USA), using an Ultra-Turrax® homogenizer and concentration and quality of the RNA obtained was determined using a NanoDrop equipment (NanoDrop Technologies, Wilmington, DE, USA) and an Agilent bioanalyzer device (Agilent Technologies, Palo Alto, CA, USA). First-strand cDNA synthesis was carried out with Superscript II (Invitrogen Life Technologies, Paisley, UK).

The qPCR assessment included genes involved in antioxidant homeostasis, vascularization and prenatal growth (*SOD1*, *CAT*, *HIF1A*, *VEGFA*, *NOS2*, *IGF1* and *UCP2*) and it was performed as previously described for our group [50]. Stability of endogenous genes was tested with Genorm software [51] and finally, *ACTB* and *B2M* genes were employed for normalization. Data for primers (designed using Primer Select software; DNASTAR, Madison, WI, USA), amplicon lengths and PCR efficiencies are indicated in Table 6.

### 4.8. Evaluation of Fetal DNA Methylation

The assessment of DNA methylation was performed in whole blood after extraction of genomic DNA (QIAamp DNA Blood Mini Kit; Qiagen, Valencia, CA) analysis by capillary electrophoresis as described previously [33]. The percentage of methylated to total cytosine (mCyt/tCyt) was calculated using the formula: µmol mCyt/(µmol mCyt + µmol Cyt) * 100. The inter-assay CV for mCyt/tCyt measurements was 3.3%.

The role of methylneogenesis for donation of methyl groups and DNA-remethylation through the folate pathway was assessed by determining the ratio of serine to glycine at fetal plasma. The conversion of serine to glycine produces 5,10-methylenetetrahydrofolate (CH2-THF), which facilitates the generation of S-adenosylmethionine (SAM), the methyl donor for DNA methylation reactions.

### 4.9. Statistical Analyses

Data were analyzed using SPSS 22.0 (IBM, NY). T-student tests were used to assess the effects of maternal diet (control vs. treated) on litter size and distribution of sexes. Two-way ANOVA and Duncan’s post-hoc test were used to assess possible effects of diet (control vs. treated) and fetal sex (female vs. male) on developmental traits, metabolic and antioxidant status, and DNA methylation, considering the sow as the experimental unit for avoiding possible biases from litter size.

Statistical analysis of gene expression data was carried out following the method proposed by Steibel et al. in 2009 [52] and adapted to our laboratory [50].

Four different groups were fitted to such a model: hidroxitirosol effects (two levels: C and HTX) in two sexes and the four combinations of the two treatments and two sexes. To test differences or interaction between classes in the expression rate of genes of interest (diffTG) normalized by the endogenous genes, different contrasts were performed between the appropriate estimates of TG levels. The significance of diffTG estimates was determined with the t statistic. Correlation among candidate genes’ expression quantities (normalized values) and with fetal weight measures was calculated in R environment.

All results were expressed as mean ± SEM and statistical significance was accepted from *p* < 0.05, while *P*-values between 0.05 and 0.09 were considered to indicate a tendency.

## 5. Conclusions

Supplementation of maternal diet with hydroxytyrosol during pregnancy improves fetal antioxidant status and glucose metabolism in a sex-dependent manner, in which males were favored. However, these male fetuses exposed to maternal hydroxytyrosol supplementation showed worse developmental patterns when compared to both control counterparts and female littermates. Further studies need to elucidate if this event is a consequence of hydroxytyrosol supplementation or, on the contrary, if the effects of hydroxytyrosol on antioxidant and metabolic status are more evident in the fetuses at higher risk of growth retardation. Finally, no major deleterious effects of hydroxytyrosol supplementation on DNA methylation and constriction of the ductus arteriosus were found.

## Figures and Tables

**Table 1:** Maternal features at day of sampling (Day 100 of pregnancy). Mean values (±SEM) in treated (Hydroxytyrosol; Group HTX) and control sows (Group C). TEAC accounts for Trolox Equivalent Antioxidant Capacity, HDL-c for high-density lipoproteins cholesterol and LDL-c for low-density lipoproteins cholesterol).

Maternal Features	HTX	C
Body-Weight (Kg)	94.7 ± 3.8	98.6 ± 6.9
Back-fat depth (cm)	1.9 ± 0.1	2.2 ± 0.3
TEAC (mmol/L)	5.6 ± 0.1	5.6 ± 0.1
Glutathione (µmol/L)	383.4 ± 49.8	388.3 ± 108.9
Glycine (µmol/L)	805.5 ± 25.5	757.1 ± 34.7
Taurine (µmol/L)	106.2 ± 23.1	83.3 ± 9.4
Serine (µmol/L)	154.2 ± 19.6	140.5 ± 21.6
Glucose (mg/dL)	97.5 ± 3.9	96.8 ± 3.7
Fructosamine (mg/dL)	321.7 ± 22.4	308.1 ± 15.5
Triglycerides (mg/dL)	35.3 ± 3.6	33.1 ± 2.9
Total cholesterol (mg/dL)	75.7 ± 7.5	66.6 ± 5.0
HDL-cholesterol (mg/dL)	28.4 ± 1.9	25.6 ± 1.1
LDL-cholesterol (mg/dL)	38.5 ± 4.6	33.2 ± 2.9

**Table 2:** Fetal antioxidant status at day of sampling (Day 100 of pregnancy). Mean values (±SEM) and differences for sexes in treated (Hydroxytyrosol; Group HTX) and control fetuses (Group C). TEAC accounts for Trolox Equivalent Antioxidant Capacity. Superscript letters indicate significant differences between groups: a ≠ b: *p* < 0.9, c ≠ d: *p* < 0.05, e ≠ f: *p* < 0.01, g ≠ h: *p* < 0.005.

Parameter	HTX			C		
	Mean	Female	Male	Mean	Female	Male
TEAC (mmol/L)	2.2 ± 0.05 ^g^	2.1 ± 0.07	2.3 ± 0.08	2.1 ± 0.04 ^h^	2.2 ± 0.06	2.1 ± 0.07
Glutathione (µmol/L)	390.2 ± 15.9 ^a^	389.7 ± 19.1	395.7 ± 23.5	354.7 ± 17.9 ^a^	374.6 ± 25.6	338.4 ± 38.7
Glycine (µmol/L)	691.9 ± 32.1 ^c^	718.3 ± 14.1	661.3 ± 17.4	770.5 ± 26.6 ^d^	797.3 ± 50.8	730.4 ± 62.3
Serine (µmol/L)	262.9 ± 8.1	281.4 ± 10.8	237.9 ± 9.5	291.1 ± 17.7	306.1 ± 28.7	271.7 ± 16.4
Taurine (µmol/L)	304.1 ± 21.5	311.7 ± 19.3	286.9 ± 23.8	320.9 ± 17.8	314.1 ± 31.7	320.6 ± 38.2

**Table 3:** Fetal development at day of sampling (Day 100 of pregnancy). Mean values (±SEM) and differences for sexes in treated (Hydroxytyrosol; Group HTX) and control fetuses (Group C). CRL accounts for Crown-rump Length, ONL for Occipito-Nasal Length, BPD for Biparietal Diameter, TC for Thoracic Circumference and AC for Abdominal Circumference. Superscript letters indicate significant differences between groups: a ≠ b: *p* < 0.9, c ≠ d: *p* < 0.05, e ≠ f: *p* < 0.01, g ≠ h: *p* < 0.005.

Parameter	HTX			C		
	Mean	Female	Male	Mean	Female	Male
CRL	21.3 ± 0.2	21.3 ± 0.3	21.6 ± 0.3	21.4 ± 0.2	21.2 ± 0.4	21.8 ± 0.4
ONL	11.8 ± 0.1	11.7 ± 0.1	11.6 ± 0.2	11.4 ± 0.1	11.4 ± 0.1	11.5 ± 0.2
BPD	4.1 ± 0.1 ^g^	4.1 ± 0.1	4.0 ± 0.1	4.2 ± 0.1 ^h^	4.2 ± 0.1	4.3 ± 0.1
TC	17.1 ± 0.2	17.1 ± 0.2	16.9 ± 0.4	17.5 ± 0.2	17.2 ± 0.4	18.2 ± 0.3
AC	14.1 ± 0.3	14.1 ± 0.3	13.9 ± 0.4	14.3 ± 0.2	14.2 ± 0.4	14.4 ± 0.3
Body-weight	668.3 ± 18.4 ^e^	656.1 ± 21.0	667.4 ± 30.3	736.3 ± 15.5 ^f^	702.2 ± 24.3	765.3 ± 26.9
Viscerae	102.5 ± 2.8 ^c^	104.5 ± 3.7	99.8 ± 4.5	114.5 ± 3.7 ^d^	113.1 ± 4.5	116.3 ± 6.2
Brain	25.4 ± 0.6	25.8 ± 0.3	24.8 ± 1.5	25.4 ± 0.3	25.4 ± 0.4	25.4 ± 0.4
Heart	6.4 ± 0.2	6.3 ± 0.3	6.5 ± 0.3	6.7 ± 0.2	6.5 ± 0.3	7.0 ± 0.3
Lungs	22.6 ± 0.8 ^c^	22.3 ± 0.8	22.9 ± 1.5	24.9 ± 0.8 ^d^	23.7 ± 1.0	26.4 ± 1.0
Liver	19.2 ± 0.6 ^c^	19.3 ± 0.7	18.9 ± 1.1	20.9 ± 0.6 ^d^	20.2 ± 0.8	21.8 ± 0.8
Kidneys	6.2 ± 0.2	6.2 ± 0.2	6.1 ± 0.3	6.5 ± 0.2	6.5 ± 0.3	6.7 ± 0.3
Intestine	27.7 ± 0.9 ^g^	27.9 ± 1.2	27.3 ± 1.6	32.0 ± 1.1 ^h^	31.3 ± 1.7	32.9 ± 1.3
Pancreas	0.8 ± 0.01 ^c^	0.8 ± 0.01	0.7 ± 0.01	0.9 ± 0.01 ^d^	0.9 ± 0.1	0.9 ± 0.1
Spleen	1.3 ± 0.1	1.2 ± 0.1	1.3 ± 0.1	1.3 ± 0.07	1.3 ± 0.1	1.4 ± 0.1
Adrenal	0.1 ± 0.01	0.1 ± 0.01	0.1 ± 0.01	0.1 ± 0.01	0.1 ± 0.01	0.1 ± 0.01
Placenta	249.3 ± 10.3	237.6 ± 10.7	265.4 ± 19.1	271.8 ± 8.9	265.3 ± 11.5	280.2 ± 14.0

**Table 4:** Fetal muscle fibers at day of sampling (Day 100 of pregnancy). Mean values (±SEM) and differences for sexes for the number of secondary fibers (F2) and the area of the muscle fiber in treated (Hydroxytyrosol; Group HTX) and control fetuses (Group C).

Parameter	HTX			C		
	Mean	Female	Male	Mean	Female	Male
F2	30.1 ± 0.7	29.9 ± 1.0	31.8 ± 0.8	29.7 ± 0.8	32.0 ± 0.9	25.9 ± 1.4
Area	9.8 ± 0.3	9.6 ± 0.5	10.0 ± 0.4	10.4 ± 0.5	10.9 ± 0.6	7.9 ± 0.4

**Table 5:** Fetal metabolic status at day of sampling (Day 100 of pregnancy). Mean values (±SEM) and differences for sexes in treated (Hydroxytyrosol; Group HTX) and control fetuses (Group C). HDL-c for high-density lipoproteins cholesterol and LDL-c for low-density lipoproteins cholesterol). Superscript letters indicate significant differences between groups: a ≠ b: *p* < 0.9, c ≠ d: *p* < 0.05, e ≠ f: *p* < 0.01, g ≠ h: *p* < 0.005.

Parameter	HTX			C		
	Mean	Female	Male	Mean	Female	Male
Glucose (mg/dL)	123.6 ± 14.0 ^c^	133.2 ± 20.2	110.5 ± 18.4	177.6 ± 19.8 ^d^	200.2 ± 27.8	148.2 ± 27.0
Fructosamine (mg/dL)	187.2 ± 3.5 ^e^	183.6 ± 4.5	192.1 ± 5.4	167.5 ± 6.0 ^f^	174.5 ± 6.8	158.3 ± 10.5
Triglycerides (mg/dL)	47.6 ± 1.4	47.6 ± 1.8	47.5 ± 2.1	52.5 ± 2.6	53.6 ± 3.4	50.9 ± 3.9
Total Cholesterol (mg/dL)	60.3 ± 1.7	59.7 ± 2.2	61.0 ± 2.9	59.6 ± 2.0	59.6 ± 2.4	59.7 ± 3.4
HDL-c (mg/dL)	20.9 ± 0.5	21.1 ± 0.6	20.7 ± 0.8	22.6 ± 2.8	20.2 ± 0.6	25.6 ± 6.4
LDL-c (mg/dL)	28.8 ± 1.1	28.5 ± 1.5	29.2 ± 1.8	31.3 ± 1.1	30.8 ± 1.5	32.0 ± 1.7

**Table 6:** Primer design for qPCR and PCR efficiencies.

Gene Symbol	Gene Name	Reference Sequence ID	Primer Sequences	Amplicon Length (bp)	Efficiency (%)
*CAT*	Catalase	NM_214301.2	TGGCCCCATGTGCTTTCA GGCGTTTCCTCTCCTCCTCAT	209	83.75
*SOD1*	Superoxide dismutase 1	NM_001190422	GTGACTGCTGGCAAAGATGGTGTG TTTCCCGTCTTTGTACTTTCTTCA	158	89.75
*HIF1A*	Hypoxia inducible factor 1 subunit alpha	NM_001123124.1	GGCGGCGCGAACGACAAGA CACACGCAAATAGCTGATGGTAAG	195	93.8
*NOS2*	Nitric oxide synthase 2	XM_013981169.2	TGGGGCAGCGGGATGACTTTC CACCCTGGCCAGATGTTCCTC	231	77.05
*VEFGA*	Vascular endothelial growth factor A	NM_214084.1	CATCTTCAAGCCGTCCTGTGT ATTTTCTTGCCTCGCTCTATCTTT	211	92.1
*IGF1*	Insulin like growth factor 1	XM_005664199.3	TGCGGAGACAGGGGCTTTTATTTC CCTTGGGCATGTCCGTGTGG	199	88.1
*UCP2*	Uncoupling protein 2	ENSSSCG00000014833	CCCTGCGGCCCGGACACATAG GACGCCTCCACTCAGCAGCAAGAC	217	86.25
*ACTB*	Beta-actin	XM_003124280.4	TCTGGCACCACACCTTCT GATCTGGGTCATCTTCTCAC	114	89.35
*B2M*	Beta-2-microglobulin	NM_213978.1	TTCACACCGCTCCAGTAG CCAGATACATAGCAGTTCAGG	166	83
